# 

**Published:** 1876-12

**Authors:** 


### TABLE OF CONTENTS.

ORIGINAL COMMUNICATIONS—

A New Medicine—"Seven Springs" "Iron and Alum Mass," or "Compound Ferruginous Mass." By W. F. Barr, M.D., of Abingdon, Va............ 705
Post Partem Hemorrhage. By John W. Booth, M.D..... .................... 712
Physiological Antagonism in the Treatment of Fever. By E. T. Easley, A.M., M.D., Little Rock, Ark............... .................................. 721
Cypripedium in Amenorrhœa and Dysmenorrhœa. By G. H. Batte, M.D., of Virginia.............................................................. 726
Hæmaturia in Quinine. By G. D. Hodge, M.D., of Arkansas................ 728
The Influence of Quinine in Parturition. By D. P. Duncan, M.D............ 729

EXTRACTS AND GLEANIEGS—

Bronchial Catarrh—731. Remedy for Dysmenorrhœa and Consequent Sterility—732. Picric Acid for Sore Nipples—733. Nitrite of Amyl—734. Treatment of Prolapsus Recti—734. Treatment of Angina Pectoris—735. Treatment of Syphilitic Buboes by the Parenchymatous Injection of Iodide of Potassium—735. On Juniper Fumigations in the Treatment of Skin Diseases—736. Injection of Quinine in Gonorrhœa—737. The Treatment of Enuresis—737. Case of Tapeworm Successfully Treated—738. Tape Worm—739. Solution of Salicylic Acid—739. Nitrate of Lead as a Local Agent in Erysipelas—740. To Check Colliquative Sweating—740. Poison-Oak Eruption—741. Treatment of Chronic Diseases of the Stomach by Alkaline Injections—741. Treatment of Polyuria—742. Treatment of Diphtheria—742. On the Treatment of Chorea—743. Treatment of Fissured and Ulcerated Nipples—743 Injections of Chloral in Hydrophobia—744. To Prevent the Troublesome Perspiration of the Feet—744. Sulphate of Cinchonidia—745. Lemon Tonic—745. How to Decide the Question of Operation in a Case of Perityphlitis—746. Stokes' Expectorant Mixture—746. Capiliary Puncture of the Intestines in Tympanites—747. Topical Treatment of Chronic Dysentery—747. Chloral as an Application to Ulcers—748. Ulceration of the Frœnum Linguœ in Whooping Cough—748. Restoratian of Milk-Supply—748. Poisoning by Oil of Red Cedar—749. Formation of Epidermis by the Transplanting of Hairs—749. On the Use of Xanthium Spinosum in the Treatment of Hydrophobia—750. Vomiting of Pregnancy Cured by Hyoscyamia—750. A New Appliance for Bloodless Operations—751. Prevention of Miscarriage by Hypordermic Injections of Morphia—751. Fetid Feet—751. Mist. Bumstead—751. Strong Solution of Salicylic Acid—Formula of Solution—752. Daturia as a Substitute for Atropia—752. Croton Oil Paint in Pelvic Cellulitis—7[illegible]. Chronic Pneumonia of the Apex in Children—753. Diphtheria Treated with Sulphate of Iron—754. Case of Constitutional Hemorrhage—755. Diphtheritic Sore-throat—756. Treatment of Fractures—756. Bromide of Lithium—756. Treatment of Superficial Varices—756. Liquid Bismuth or Elixir of Bismuth—757. Condensed Milk as Food for Children—757. Acne—757. Nitrite of Amyl in Hiccough—758. Incontinence of Urine—758. Post Partem Hemorrhages—758. Pills for Obstinate Neuralgia—758. To Remove Foreign Substances from the Ear—758. Hydrastin Canadensis in Gleet—750. Remedy for Dandruff—759. Extraofct Beef—760. Chloral Plaster for Neuralgia, etc—760. Mist. Biniodidi—760. Porrigo Decalvans—760. Pathognomonic Symptom of Cerebro-spinal Meningitis—760. Bicarbonate of Soda in Suspression of Urine—761. Treatment of Capillary Nœvus—761. Antidote to Strychnia—761. Mist. Diarrhœa Ruschenberger, B. & C. H.—761. For Burns—761. Chloral Plaster—762. Syrup of Salicylic Acid—762. Chlorate of Potassium in Chronic Gastro-Intestinal Catarrh—762. Toothache Remedy—762. Phosphorus—762. Cincho-Quinine, Strychnine and Iron—763. Nerve Stretching in Petanus—763. Poisoning by Carbolic Acid—763. Antidote to Strychnia—763. Scrofulous Sores—763. For Whooping Cough—763.

EDITORIAL AND MISCELLANEOUS—

Close of the Volume.................................................. 764
Suggestions from a Co-laborer........................................ 764
Comments on Co-laborer.............................................. 765
Retrospective Glances................................................ 766
Special Notice........................................................ 768
To Our Advertising Agents........................................... 769
Vick's Floral Guide................................................... 769
The Sunny South...................................................... 770

